# Mesenchymal Stem Cells and Their Derived Products in Ageing and Diseases

**DOI:** 10.3390/ijms25136979

**Published:** 2024-06-26

**Authors:** Francisco J. Vizoso, Luis A. Costa, Noemi Eiro

**Affiliations:** Research Unit, Fundación Hospital de Jove, Avda. Eduardo Castro, 161, 33920 Gijón, Spain

Despite the enormous efforts of the pharmaceutical industry in the generation of new drugs (55 new ones last year) [1], around 40% of the world’s population still suffer from inflammatory and/or degenerative diseases for which there is no adequate treatment. In these pathological situations, the lack of adequate tissue homeostasis will lead to irremediable tissue loss. Moreover, this scenario is exacerbated by the progressive ageing of the population, contributing significantly to the imperative need for new therapeutic alternatives in order to increase the quality of life in face of the onset of associated diseases [2]. On the other hand, the growing knowledge derived from omics (proteomics, genomics or metagenomics) on the multifactorial nature of diseases and their complex pathological processes [3], is placing us, in many cases, before a very complex therapeutic challenge, which is difficult to address under the perspective of the classic dogma of ‘one disease, one therapeutic target’. So perhaps, the time has come to do something different—why not try to reproduce the tissue homeostatic mechanisms that mesenchymal stem cells (MSCs) carry out under physiological conditions?

MSCs are known to have regenerative, anti-inflammatory, immunoregulatory, anti-oxidative stress, anti-fibrotic, anti-microbial, and anti-tumour effects [4]. There is increasing evidence that MSCs carry out these actions in a paracrine manner through the secretion of cytokines and growth factors [5,6]. In addition, more recently, the effect of extracellular vesicles, especially exosomes (nanoparticles, 70–150 nm in size, surrounded by a membrane filled with bioactive particles), which, when released by MSCs, reproduce their biological effects in the bodily context of cell signalling, is gaining strength [7]. Interestingly, evidence is now also emerging of a link between diseases such as lupus, rheumatoid arthritis, diabetes, psoriasis, and early ageing syndromes, with MSC depletion and/or dysfunction [8]. This suggests that MSC transplantation could positively influence the natural history of many diseases. However, although there are many data indicating a favourable safety profile for the use of intravenous MSC [9], MSC transplantation has limitations that represent a barrier to widespread use of this type of cell therapy. These drawbacks include the reduced half-life of the cells once administered; their ageing with loss of putative potency; potential embolus formation; cancerous transformation, which can never be ruled out; transmission of infections to the host; as well as various technical aspects of logistics and economic costs that hinder the generalisation of this type of therapy to the population [10].

A strategic alternative could lie in the development of a cell-free regenerative therapy using products derived from the secretome obtained during cell cultures [8]. These in vitro procedures are favoured by the high replicative rate of MSC. Furthermore, there is bioreactor technology that allows us to establish favourable cultivation conditions in pursuit of mass production and improvement in the MSC-derived secretome [11]. This new technical strategy not only avoids the drawbacks of cell therapy based on MSC transplantation, but also offers advantages such as a better evaluation of the product in terms of safety, dose and potency, analogous to conventional therapeutic agents, easier storage and, ultimately, a more economical and practical approach for clinical use.

Moreover, among the microvesicles that make up the MSC secretome are exosomes which offer added advantages, such as a longer half-life in the bloodstream, the possibility of preloading with therapeutic products, their ability to cross the blood–brain barrier, their tropism towards inflamed tissues and tumours, and their ability to release bioactive substances of various kinds into target tissues [12,13]. Thus, all these technological innovations place us at the dawn of a new medicine with great therapeutic possibilities, but also with new challenges [14].

Among the challenges, opportunities, and goals of this new medicine based on mesenchymal/medicinal stem/signalling cells and their derived products are the following: (i) the appropriate choice of the type of MSC, depending on their biological niche and the biological characteristics of the donor, for each future application [8]; (ii) the controlled and reproducible production and control of the potency of their secretome-derived products; (iii) the possibility of influencing the quality of MSCs’ secretome through the use of preconditioning techniques and/or genetic manipulation; (iv) the opportunity to combine these biological products with new biomaterials, such as thermolabile hydrogels, allowing for their more optimal tissue integration and/or biodistribution [15]; (v) the potential use of MSC-derived extracellular vesicles as biological carriers of exosomes for drug delivery in cancer therapy [16,17,18] and other therapies [18,19]; (vi) the possibility of new and exciting clinical applications such as intranasal administration of MSC-derived exosomes for treating brain diseases [20]; and (vii) strict consideration of regulatory agency requirements. There is sufficient scientific knowledge about MSC biology and the therapeutic potential to support these goals, the appropriate technology to realise them, the legal framework to support new proposals, and pressing demand from millions of patients.
